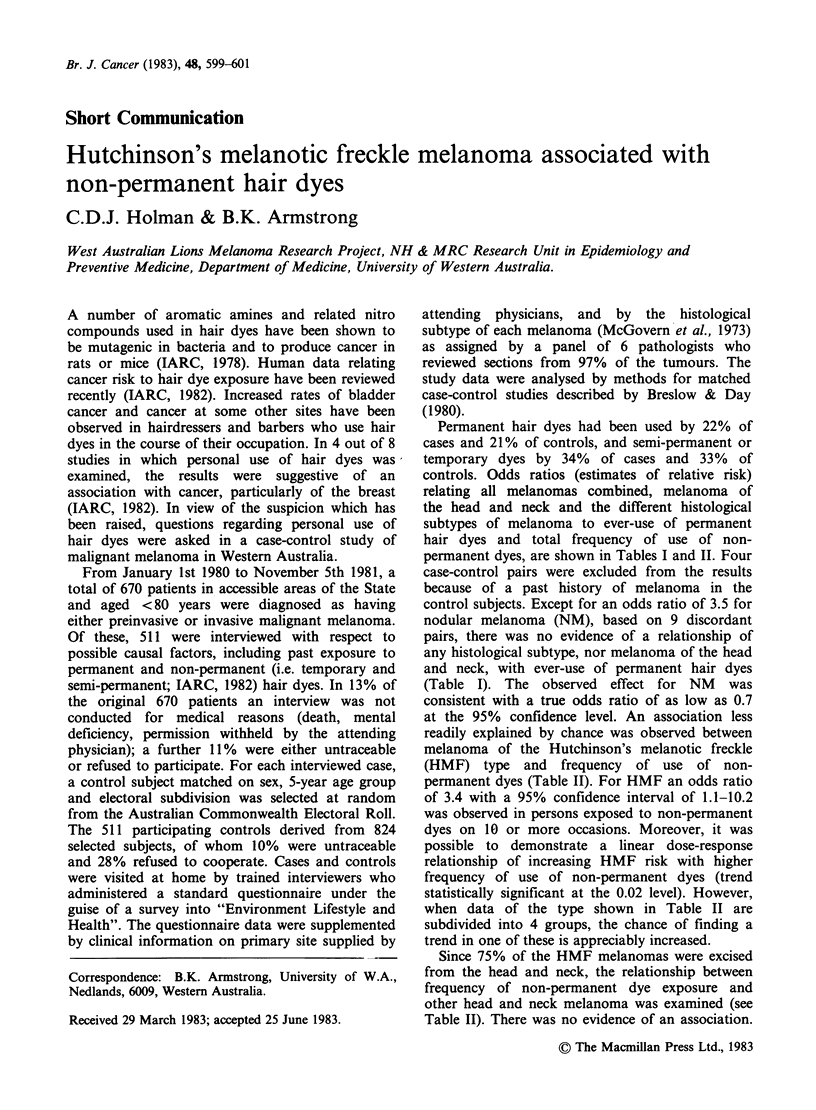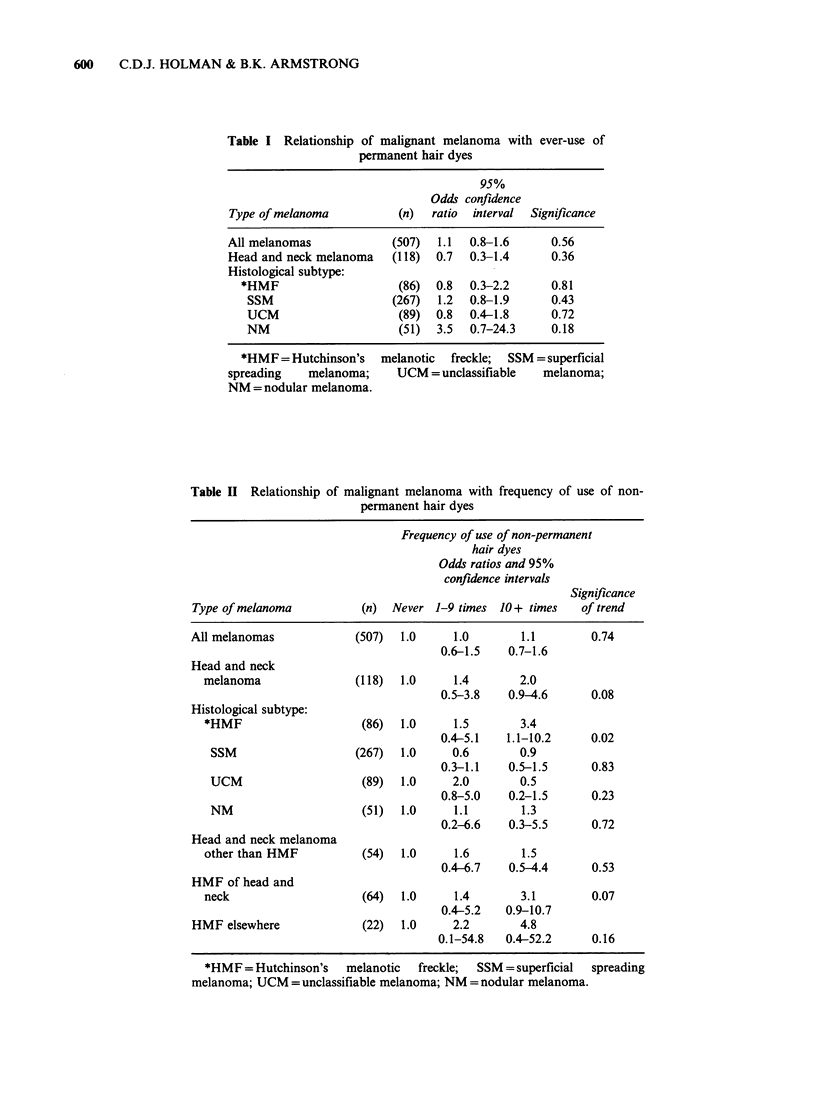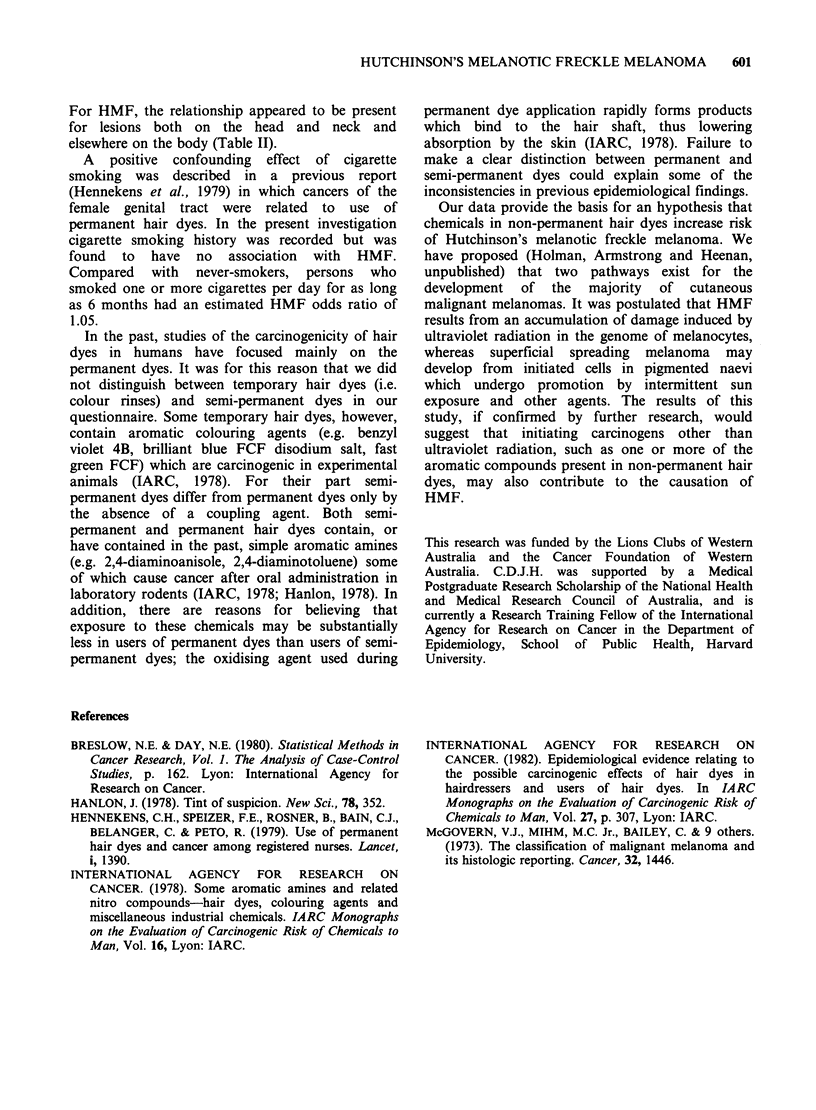# Hutchinson's melanotic freckle melanoma associated with non-permanent hair dyes.

**DOI:** 10.1038/bjc.1983.234

**Published:** 1983-10

**Authors:** C. D. Holman, B. K. Armstrong


*Br. J. Cancer* (1983), **48**, 599–601

### Short Communication

### Hutchinson's melanotic freckle melanoma associated with non-permanent hair dyes

C.D.J. Holman & B.K. Armstrong

*West Australian Lions Melanoma Research Project, NH & MRC Research Unit in Epidemiology and Preventive Medicine, Department of Medicine, University of Western Australia.*

A number of aromatic amines and related nitro compounds used in hair dyes have been shown to be mutagenic in bacteria and to produce cancer in rats or mice (IARC, 1978). Human data relating cancer risk to hair dye exposure have been reviewed recently (IARC, 1982). Increased rates of bladder cancer and cancer at some other sites have been observed in hairdressers and barbers who use hair dyes in the course of their occupation. In 4 out of 8 studies in which personal use of hair dyes was examined, the results were suggestive of an association with cancer, particularly of the breast (IARC, 1982). In view of the suspicion which has been raised, questions regarding personal use of hair dyes were asked in a case-control study of malignant melanoma in Western Australia.

From January 1st 1980 to November 5th 1981, a total of 670 patients in accessible areas of the State and aged <80 years were diagnosed as having either preinvasive or invasive malignant melanoma. Of these, 511 were interviewed with respect to possible causal factors, including past exposure to permanent and non-permanent (i.e. temporary and semi-permanent; IARC, 1982) hair dyes. In 13% of the original 670 patients an interview was not conducted for medical reasons (death, mental deficiency, permission withheld by the attending physician); a further 11% were either untraceable or refused to participate. For each interviewed case, a control subject matched on sex, 5-year age group and electoral subdivision was selected at random from the Australian Commonwealth Electoral Roll. The 511 participating controls derived from 824 selected subjects, of whom 10% were untraceable and 28% refused to cooperate. Cases and controls were visited at home by trained interviewers who administered a standard questionnaire under the guise of a survey into "Environment Lifestyle and Health". The questionnaire data were supplemented by clinical information on primary site supplied by attending physicians, and by the histological subtype of each melanoma (McGovern *et al.*, 1973) as assigned by a panel of 6 pathologists who reviewed sections from 97% of the tumours. The study data were analysed by methods for matched case-control studies described by Breslow & Day (1980).

Permanent hair dyes had been used by 22% of cases and 21% of controls, and semi-permanent or temporary dyes by 34% of cases and 33% of controls. Odds ratios (estimates of relative risk) relating all melanomas combined, melanoma of the head and neck and the different histological subtypes of melanoma to ever-use of permanent hair dyes and total frequency of use of non-permanent dyes, are shown in Tables I and II. Four case-control pairs were excluded from the results because of a past history of melanoma in the control subjects. Except for an odds ratio of 3.5 for nodular melanoma (NM), based on 9 discordant pairs, there was no evidence of a relationship of any histological subtype, nor melanoma of the head and neck, with ever-use of permanent hair dyes (Table I). The observed effect for NM was consistent with a true odds ratio of as low as 0.7 at the 95% confidence level. An association less readily explained by chance was observed between melanoma of the Hutchinson's melanotic freckle (HMF) type and frequency of use of non-permanent dyes (Table II). For HMF an odds ratio of 3.4 with a 95% confidence interval of 1.1–10.2 was observed in persons exposed to non-permanent dyes on 10 or more occasions. Moreover, it was possible to demonstrate a linear dose-response relationship of increasing HMF risk with higher frequency of use of non-permanent dyes (trend statistically significant at the 0.02 level). However, when data of the type shown in Table II are subdivided into 4 groups, the chance of finding a trend in one of these is appreciably increased.

Since 75% of the HMF melanomas were excised from the head and neck, the relationship between frequency of non-permanent dye exposure and other head and neck melanoma was examined (see Table II). There was no evidence of an association.

Correspondence: B.K. Armstrong, University of W.A., Nedlands, 6009, Western Australia.

Received 29 March 1983; accepted 25 June 1983.

© The Macmillan Press Ltd., 1983

**Table I** Relationship of malignant melanoma with ever-use of permanent hair dyes

| *Type of melanoma* | (*n*) | *Odds ratio* | *95% confidence interval* | *Significance* |
|---|---|---|---|---|
| All melanomas | (507) | 1.1 | 0.8–1.6 | 0.56 |
| Head and neck melanoma | (118) | 0.7 | 0.3–1.4 | 0.36 |
| Histological subtype: | | | | |
| *HMF | (86) | 0.8 | 0.3–2.2 | 0.81 |
| SSM | (267) | 1.2 | 0.8–1.9 | 0.43 |
| UCM | (89) | 0.8 | 0.4–1.8 | 0.72 |
| NM | (51) | 3.5 | 0.7–24.3 | 0.18 |

*HMF = Hutchinson's melanotic freckle; SSM = superficial spreading melanoma; UCM = unclassifiable melanoma; NM = nodular melanoma.

**Table II** Relationship of malignant melanoma with frequency of use of non-permanent hair dyes

| *Type of melanoma* | (*n*) | *Frequency of use of non-permanent hair dyes Odds ratios and 95% confidence intervals* Never | 1–9 times | 10+ times | *Significance of trend* |
|---|---|---|---|---|---|
| All melanomas | (507) | 1.0 | 1.0 | 1.1 | 0.74 |
| | | | 0.6–1.5 | 0.7–1.6 | |
| Head and neck melanoma | (118) | 1.0 | 1.4 | 2.0 | |
| | | | 0.5–3.8 | 0.9–4.6 | 0.08 |
| Histological subtype: | | | | | |
| *HMF | (86) | 1.0 | 1.5 | 3.4 | |
| | | | 0.4–5.1 | 1.1–10.2 | 0.02 |
| SSM | (267) | 1.0 | 0.6 | 0.9 | |
| | | | 0.3–1.1 | 0.5–1.5 | 0.83 |
| UCM | (89) | 1.0 | 2.0 | 0.5 | |
| | | | 0.8–5.0 | 0.2–1.5 | 0.23 |
| NM | (51) | 1.0 | 1.1 | 1.3 | |
| | | | 0.2–6.6 | 0.3–5.5 | 0.72 |
| Head and neck melanoma other than HMF | (54) | 1.0 | 1.6 | 1.5 | |
| | | | 0.4–6.7 | 0.5–4.4 | 0.53 |
| HMF of head and neck | (64) | 1.0 | 1.4 | 3.1 | 0.07 |
| | | | 0.4–5.2 | 0.9–10.7 | |
| HMF elsewhere | (22) | 1.0 | 2.2 | 4.8 | |
| | | | 0.1–54.8 | 0.4–52.2 | 0.16 |

*HMF = Hutchinson's melanotic freckle; SSM = superficial spreading melanoma; UCM = unclassifiable melanoma; NM = nodular melanoma.

For HMF, the relationship appeared to be present for lesions both on the head and neck and elsewhere on the body (Table II).

A positive confounding effect of cigarette smoking was described in a previous report (Hennekens *et al.*, 1979) in which cancers of the female genital tract were related to use of permanent hair dyes. In the present investigation cigarette smoking history was recorded but was found to have no association with HMF. Compared with never-smokers, persons who smoked one or more cigarettes per day for as long as 6 months had an estimated HMF odds ratio of 1.05.

In the past, studies of the carcinogenicity of hair dyes in humans have focused mainly on the permanent dyes. It was for this reason that we did not distinguish between temporary hair dyes (i.e. colour rinses) and semi-permanent dyes in our questionnaire. Some temporary hair dyes, however, contain aromatic colouring agents (e.g. benzyl violet 4B, brilliant blue FCF disodium salt, fast green FCF) which are carcinogenic in experimental animals (IARC, 1978). For their part semi-permanent dyes differ from permanent dyes only by the absence of a coupling agent. Both semi-permanent and permanent hair dyes contain, or have contained in the past, simple aromatic amines (e.g. 2,4-diaminoanisole, 2,4-diaminotoluene) some of which cause cancer after oral administration in laboratory rodents (IARC, 1978; Hanlon, 1978). In addition, there are reasons for believing that exposure to these chemicals may be substantially less in users of permanent dyes than users of semi-permanent dyes; the oxidising agent used during permanent dye application rapidly forms products which bind to the hair shaft, thus lowering absorption by the skin (IARC, 1978). Failure to make a clear distinction between permanent and semi-permanent dyes could explain some of the inconsistencies in previous epidemiological findings.

Our data provide the basis for an hypothesis that chemicals in non-permanent hair dyes increase risk of Hutchinson's melanotic freckle melanoma. We have proposed (Holman, Armstrong and Heenan, unpublished) that two pathways exist for the development of the majority of cutaneous malignant melanomas. It was postulated that HMF results from an accumulation of damage induced by ultraviolet radiation in the genome of melanocytes, whereas superficial spreading melanoma may develop from initiated cells in pigmented naevi which undergo promotion by intermittent sun exposure and other agents. The results of this study, if confirmed by further research, would suggest that initiating carcinogens other than ultraviolet radiation, such as one or more of the aromatic compounds present in non-permanent hair dyes, may also contribute to the causation of HMF.

This research was funded by the Lions Clubs of Western Australia and the Cancer Foundation of Western Australia. C.D.J.H. was supported by a Medical Postgraduate Research Scholarship of the National Health and Medical Research Council of Australia, and is currently a Research Training Fellow of the International Agency for Research on Cancer in the Department of Epidemiology, School of Public Health, Harvard University.

### References

BRESLOW, N.E. & DAY, N.E. (1980). *Statistical Methods in Cancer Research, Vol. 1. The Analysis of Case-Control Studies*, p. 162. Lyon: International Agency for Research on Cancer.

HANLON, J. (1978). Tint of suspicion. *New Sci.*, **78**, 352.

HENNEKENS, C.H., SPEIZER, F.E., ROSNER, B., BAIN, C.J., BELANGER, C. & PETO, R. (1979). Use of permanent hair dyes and cancer among registered nurses. *Lancet*, **i**, 1390.

INTERNATIONAL AGENCY FOR RESEARCH ON CANCER. (1978). Some aromatic amines and related nitro compounds—hair dyes, colouring agents and miscellaneous industrial chemicals. *IARC Monographs on the Evaluation of Carcinogenic Risk of Chemicals to Man*, Vol. **16**, Lyon: IARC.

INTERNATIONAL AGENCY FOR RESEARCH ON CANCER. (1982). Epidemiological evidence relating to the possible carcinogenic effects of hair dyes in hairdressers and users of hair dyes. In *IARC Monographs on the Evaluation of Carcinogenic Risk of Chemicals to Man*, Vol. **27**, p. 307, Lyon: IARC.

McGOVERN, V.J., MIHM, M.C. Jr., BAILEY, C. & 9 others. (1973). The classification of malignant melanoma and its histologic reporting. *Cancer*, **32**, 1446.